# Dosimetric impact of adaptive proton therapy in head and neck cancer – A review

**DOI:** 10.1016/j.ctro.2023.100598

**Published:** 2023-02-16

**Authors:** Merle Huiskes, Eleftheria Astreinidou, Wens Kong, Sebastiaan Breedveld, Ben Heijmen, Coen Rasch

**Affiliations:** a Department of Radiation Oncology, Leiden University Medical Center, Leiden, the Netherlands; b Department of Radiotherapy, Erasmus MC Cancer Institute, University Medical Center Rotterdam, the Netherlands; c HollandPTC, Delft, the Netherlands

**Keywords:** Adaptive proton therapy, Intensity modulated proton therapy, Head and neck cancer, Dosimetric impact, Review

## Abstract

**Background:**

Intensity Modulated Proton Therapy (IMPT) in head and neck cancer (HNC) is susceptible to anatomical changes and patient set-up inaccuracies during the radiotherapy course, which can cause discrepancies between planned and delivered dose. The discrepancies can be counteracted by adaptive replanning strategies. This article reviews the observed dosimetric impact of adaptive proton therapy (APT) and the timing to perform a plan adaptation in IMPT in HNC.

**Methods:**

A literature search of articles published in PubMed/MEDLINE, EMBASE and Web of Science from January 2010 to March 2022 was performed. Among a total of 59 records assessed for possible eligibility, ten articles were included in this review.

**Results:**

Included studies reported on target coverage deterioration in IMPT plans during the RT course, which was recovered with the application of an APT approach. All APT plans showed an average improved target coverage for the high- and low-dose targets as compared to the accumulated dose on the planned plans. Dose improvements up to 2.5 Gy (3.5 %) and up to 4.0 Gy (7.1 %) in the D98 of the high- and low dose targets were observed with APT. Doses to the organs at risk (OARs) remained equal or decreased slightly after APT was applied. In the included studies, APT was largely performed once, which resulted in the largest target coverage improvement, but eventual additional APT improved the target coverage further. There is no data showing what is the most appropriate timing for APT.

**Conclusion:**

APT during IMPT for HNC patients improves target coverage. The largest improvement in target coverage was found with a single adaptive intervention, and an eventual second or more frequent APT application improved the target coverage further. Doses to the OARs remained equal or decreased slightly after applying APT. The most optimal timing for APT is yet to be determined.

## Highlights

• APT during IMPT for HNC patients improves target coverage.
• Doses to the OARs remain equal or decrease slightly after applying APT.
• The most optimal timing for APT is yet to be determined.

## Introduction

Radiotherapy (RT) is a commonly applied curative treatment option for head and neck cancer (HNC) patients. RT for HNC is challenging as organs at risk (OARs) are located close to the tumor, and conformal dose distributions are typically delivered with advanced photon therapy techniques, such as intensity modulated photon radiotherapy (IMRT).[1], [2] Since several years, intensity modulated proton therapy (IMPT) has become more widely available as an alternative treatment technique to IMRT. For a subgroup of patients, IMPT has dosimetric benefits over IMRT, reducing the dose to the surrounding healthy tissues while preserving target coverage due to its characteristic Bragg peak.[3] However, due to the steep distal dose gradient in proton beams, IMPT is more sensitive to anatomical and set-up changes during the RT course. These changes can arise from a combination of positioning errors, weight loss and tumor, OAR or lymph node shrinkage [4], and can cause a discrepancy between the planned and delivered dose, which may in turn lead to decreased tumor control and undesirable dose increase in OARs. Therefore, robust optimization methods can be applied during IMPT planning, which take multiple set-up and range scenarios into account, aiming to minimize the effect of these variations.[5] However, no anatomical variations are incorporated in robust optimization for HNC, and still a discrepancy can occur between the planned and actual delivered dose. This can be counteracted by replanning based on re-imaging, so-called adaptive radiotherapy (ART). ART aims to ensure the delivery of the prescribed dose to the target while maximally maintaining the dose constraints to OARs.

In case of slow (week-weeks) anatomy changes, such as caused by weight loss, there is no need for daily plan adaptations; adaptations can be performed offline and patient re-imaging, re-contouring, and plan (re-)optimization are performed outside the treatment room. Offline adaptive interventions can for example be triggered by anatomical changes visible in in-room images, but ART can also be applied at predefined fixed moments. Because of the required re-imaging, re-contouring and plan (re-)optimization, ART is workload- and resource intensive. Generally, it takes from a few hours to a couple of days before a new adjusted plan is clinically available [6].

In online ART, replanning is performed in-room prior to the dose delivery while the patient is positioned on the treatment couch.[7] Therefore, online ART can respond to the more rapid interfractional changes in patient anatomy, such as nasal cavity filling variations. Since online ART has many challenges, clinical application has to the best of our knowledge not yet been performed for HNC.[6].

While most research on ART is performed for photon therapy [8], for HNC proton therapy it is yet unclear which adaptive proton therapy (APT) strategies are beneficial. Also, what has not been answered yet is what is the optimal timing to perform a plan adaptation in HNC IMPT, how often this is needed, and what the effect of APT on dosimetric outcomes is. For these reasons, a review on the existing relevant literature was performed to give an overview of what is currently known, and to identify existing gaps in this knowledge. For this, the following research questions were formulated: 1) What is the impact of APT on target coverage and dose to OARs? and 2) What is the optimal frequency and timing for APT during the IMPT course?

## Methods

To identify potentially relevant documents, a literature search was performed in PubMed/MEDLINE, EMBASE and Web of Science with the following search strategy: (“Head and Neck Neoplasms”[Mesh] OR “head and neck”[tw] OR “pharyn*”[tw] OR “oropharyn*”[tw] OR “nasopharyn*”[tw] OR “hypopharyn*”[tw] OR “laryn*”[tw] OR “paranasal sinus”[tw] OR “mouth”[tw] OR “Gingival”[tw] OR “Oral Leukoplakia”[tw] OR “Lip”[tw] OR “Lips”[tw] OR “Palatal”[tw] OR “Salivary Gland*”[tw] OR “Tongue”[tw]) AND (“adapt*”[ti]) AND (“Protons”[Mesh] OR “Proton*”[tw] OR “IMPT”[tw]). The search was completed by March 17th, 2022.

To be included in the review, studies needed to report on the dosimetric outcomes (e.g., dose to targets/OARs or dose volume histograms (DVHs)) for APT strategies. Peer reviewed journal papers were included if they were published between the period of 2010 and 2022, had at least ten HNC patients included, investigated at least one APT strategy and reported the dosimetric outcomes. Studies in languages other than English and papers where only the abstract was available were excluded. Also, review papers and papers with focus on algorithm testing/mathematical development were excluded.

Titles, abstracts, and then full text of all publications were screened independently by two reviewers for potentially relevant publications and discussed afterwards for agreement.

We abstracted data on patient characteristics (e.g., HNC subsite and number of patients included), planning details (e.g., target, robustness margin), characteristics of the APT methods (e.g., online or offline), timing and frequency of the performed APT and on dosimetric parameters (e.g., clinical goals, dose to targets/OARs) from the included studies. The dosimetric results are denoted per parameter (e.g., D98, Dmean or Dmax) in percentages for the targets and in Gray (Gy) for the OARs. Studies were grouped by the investigated method of APT and the data as mentioned above was summarized in tables.

## Results

### Literature search

**Fig. 1:**
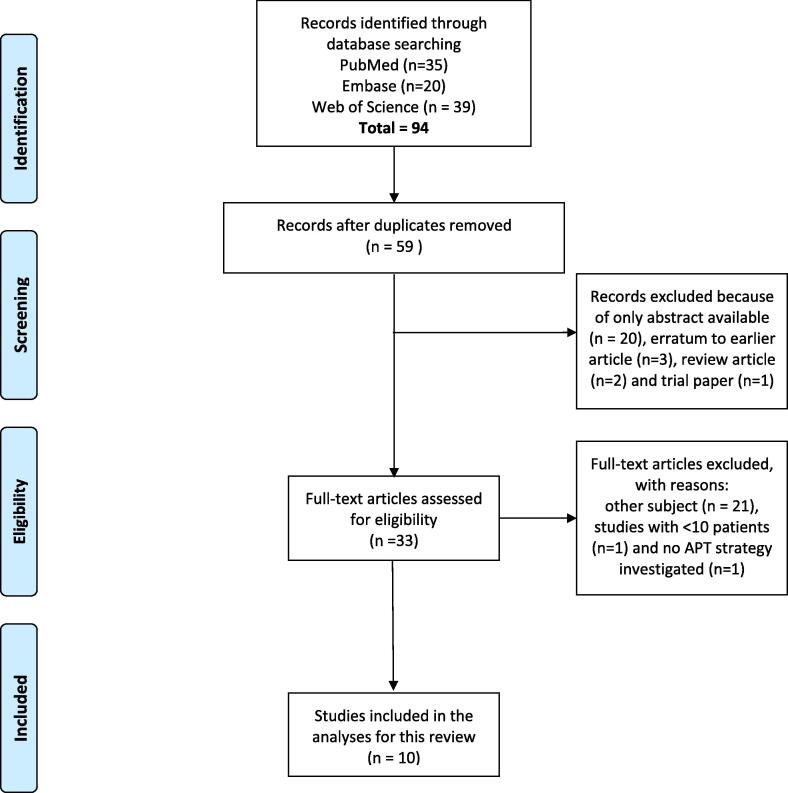
PRISMA flow diagram of the literature search.

In Fig. 1, the outcome of the search strategy is presented in a FLOW diagram. Upfront, we decided to include studies with at least 10 patients. However, since the small amount of relevant studies, we decided to also include the study by Kurz et al. [17] which investigated APT in 9 patients. Ultimately, ten studies were retained for this review. Only one of the ten included studies investigated clinical APT, [16], the other ones were simulations (Table 1). As described by Paganetti et al. [19], online adaptation plan optimization is based on in room imaging prior to the treatment, and on-line adaptation in a clinical workflow has to be completed within minutes.

**Table 1:** Overview of included studies with patient characteristics and APT strategies.

				Replanning strategy			Imaging modality		
Author (Year)	Nr of patients	Tumor site (Nr)	Target, margin	Frequency	Timing	Online/offline	Per-treatment imaging	Dose re-calculation	Clinical/simulation APT
Bobić (2021) [9]	10	OP (6), tongue (2), larynx (2)	CTV, 0 mm	Daily and weekly	–	Online	CBCT	CBCT	Simulation
Minatogawa (2021) [10]	10	NP (10)	PTV, 3 mm from CTV	Once	Fx 12–14	Offline	Repeat CT	Repeat CT	Simulation (Clinical adaptive IMRT)
Lalonde (2021) [11]	10	OP (6), tongue (2), larynx (2)	CTV, 3 mm robust	Once or daily	aRO: first two daily CBCTs	Online	CBCT	CBCT	Simulation
Nesteruk (2021) [12]	10	OC, OP, larynx	CTV, 0 mm	Daily	–	Online	CBCT	CBCT	Simulation
Yang (2020) [13]	10	NP (6), OP (4)	CTV, 3 mm robust	Once or twice	1st APT: Fx 8–14 2nd APT: 12–17 fx after 1st APT aRO: CT of 1st APT	Offline	Repeat CT	Repeat CT	Simulation (Clinical 2x APT)
Cubillos-Mesías (2019) [14]	20	HNC, subsite NA	CTV, 3 mm robust	Once	aRO: first two weekly repeat CTs	Offline	Repeat CT	Repeat CT	Simulation
Botas (2018) [15]	10	OP (5), mouth (1), tongue(1), HP (1), larynx (1), right neck (1)	CTV, 0 mm	Weekly	–	Online	CBCT	CBCT	Simulation
Wu (2017) [16]	10	OP	CTV, distal and proximal: 0 cm, lateral: 1 cm	Once	4th week	Offline	Repeat CT	Repeat CT	Clinical APT
Kurz (2016) [17]	9	Larynx (3), HP (2), NP (1), NC (3)	PTV, 5–7 mm from CTV	Once	30–50 days after planning-CT	Offline	CBCT and repeat CT	CBCT (vCT) and repeat CT	Simulation
Simone (2011) [18]	10	OP (7), SGL (2), NP (1)	PTV, 5 mm from CTV	Once	On average after 2.9 weeks	Offline	Repeat CT	Repeat CT	Simulation (Clinical adaptive IMRT)

Nr: number, HNC: Head and neck cancer, OP: oropharynx, NP: nasopharynx, HP: hypopharynx, NC: nasal cavity, SGL: supraglottic larynx, OC: oral cavity, NA: not applicable, CTV: clinical target volume, PTV: planning target volume, APT: adaptive proton therapy, Fx: fraction, aRO: anatomical robust optimization, CT: computerized tomography, CBCT: cone-beam CT, vCT: virtual CT.

Most included studies investigated the dosimetric impact of an offline APT strategy at one single moment, but also weekly and daily (online) APT frequencies were investigated in some studies. Four studies investigated an online APT strategy, which were based on simulations. Six studies investigated offline APT, five of these studies were also based on simulations, and in one study clinical APT was analyzed. A per-study overview of the different methods for APT can be found in Table 1, together with the studied patient group.

### Dosimetric results

#### Target coverage

Overall, the studies included in this review showed that target coverage in IMPT plans deteriorated below the clinical goals during the RT course, which was recovered with the application of an APT approach. All APT plans showed an average improved target coverage for the high- and low- dose targets as compared to the accumulated non-APT plans (Table 2). Average dose improvement up to 2.5 Gy (3.6 %) in D98 of the 70 Gy high-dose target was observed [12] with APT, and up to 4.0 Gy (7.1 %) for the 57 Gy low-dose target in [13].

**Table 2:** Accumulated dose to the targets, with and without APT strategy applied. Results denoted as median (min–max) or mean ± SD.

		High dose target# D98 [%]		High dose target# D2 [%]		Low dose target# D98 [%]	
Author (Year)	Total dose (low–high level [Gy])	Non-APT acc. dose	APT acc. dose	Non-APT acc. dose	APT acc. dose	Non-APT acc. dose	APT acc. dose
Bobić (2021) [9]	57–70	94.70 (92.96–98.06)	OAw: 97.61 (95.02–99.26) OAd: 98.07 (97.15–99.73)	103.48 (101.60–110.22)	OAw: 102.44 (101.91–103.47) OAd: 102.58 (102.00–103.45)	95.07 (89.76–96.39)	OAw: 97.60 (97.03–98.43) OAd: 97.97 (97.36–98.65)
Minatogawa (2021) [10]	70	NA	D95: 102.3 ± 1.1	NA	D5: 71.4 ± 0.5	NA	NA
Lalonde (2021) [11]	57–70	96.58 (91.81–97.92)	aRO: 97.47 (94.25–98.66) OA: 98.07 (97.15–99.73)	102.33 (101.08–103.61)	aRO:102.66 (101.91–104.13) OA: 102.58 (102.00–103.54)	95.06 (87.88–96.17)	aRO: 96.54 (91.44–98.53) OA: 97.97 (97.34–98.65)
Nesteruk (2021) [12]	57–70	94.5 (90.7–99.1)	98.1 (96.7–99.6)	103.2 (102.3–106.8)	102.4 (102.0–103.2)	95.2 (91.3–97.1)	98.2 (96.7–99.6)
Yang (2020) [13]	57, 63, 70	98.80 ± 1.66	2nd APT: 100.89 ± 0.47 1st APT: 100.26 ± 1.04 aRO: 100.66 ± 0.84	109.14 ± 1.69	2nd APT: 106.86 ± 0.69 1st APT: 106.80 ± 1.80 aRO: 108.86 ± 2.67	93.81 ± 5.86	2nd APT: 100.88 ± 13.58 1st APT: 99.14 ± 14.16 aRO: 100.49 ± 3.75
Cubillos-Mesías (2019) [14]	57–70	96.86 (89.16–98.91)	aRO: 97.74 (94.21–98.60)	103.46 (101.47–106.67)	aRO: 103.48 (100.57–106.01)	96.07 (88.39–99.14)	aRO: 97.17 (92.37–98.96)
Botas (2018) [15]	60	90.2 ± 8.9	Free: 97.5 ± 1.5	108.4 ± 1.7	Free: 108.0 ± 1.1	NA	NA
Wu (2017) [16]	57, 63, 70	D99: 98.59, D95: 100.21	D99: 99.16, D95: 101.1	NA	NA	D95: 100.51	D95: 109.26
Kurz (2016) [17]	50/50.4/54/54.4–56/60/61.6/64	Comparable or higher D95 as planned*	D95 comparable or mitigated* Overall, only minor differences	Overdosage in low- and high dose PTV*	Overdosage was reduced or eliminated* On average significant D2 reduction	D95 reduction of 4 Gy PTV (1 of the 2 patients)*	D95 PTV coverage almost recovered* Overall, only minor differences
Simone (2011) [18]	50, 64, 70	D100: 99.0 ± 0.7	D100: 99.4 ± 0 0.7	NA	NA	D100: 99.9 ± 0.1	D100: 99.9 ± 0.1

APT: adaptive proton therapy, acc.: accumulated, D100/99/98/95/5/2: dose to 100/99/98/95/5/2% of the volume, aRO: anatomical robust optimization, OA: online adaptation, OAw: weekly online adaptation, OAd: daily online adaptation, NA: not applicable, Free: geometric shift strategy.

* Individual results for 2 representative patients.

# Defined as the target volume with the high- and respectively low dose level prescription as stated under ‘Total dose’ column.

For the D2 of the 70 Gy high-dose target, one study [13] showed that on average the clinical constraint (<107 %) was exceeded in the cumulative planned dose, which was restored to below this goal when at least one APT was performed. Other studies did not reveal such differences for the high-dose D2 in the APT plans, see Table 2.

Looking at individual cases, several studies specifically reported on observed target underdosage [9], [11], [12], [14], [16], overdosage [17] or at least compromised target coverage [13] for multiple patients in the cumulative dose of the non-adaptive plans. For these individual patients, target coverage was reported to be restored and improved in all the studied patients after application of an APT strategy in [9], [11], [12], [17], or in nearly all (19/20) patients [14]. Study [13], [16] did not report on these individual patients after APT was applied, and only presented the average results for all patients.

#### OAR dose

Different from the targets, OAR dose changes during the RT course were not the motive to perform APT. In most of the OARs only small changes (in the order of ± 1.5 Gy) in mean- or max doses were observed during RT course in the non-APT plans, which always remained below the clinical constraints. The largest average OAR mean dose increases during RT treatment in absence of APT were observed in the ipsilateral parotid (4 Gy) and larynx (2.5 Gy) [11], but still remained below the clinical constraints as stated by the authors.

After APT was applied, which had the intention to restore target coverage as mentioned above, also an additional benefit in the OARs was observed. The doses to OARs were overall similar or lower, up to 8.8 Gy reduction in mean dose for larynx and 6.4 Gy reduction for the PCMs [11] in the APT plans were found compared to the accumulated non-APT doses. Also, a reduction of > 10 Gy in the maximum dose in the spinal cord was observed as well as a 4 Gy reduction in the brainstem [11]. The average measures and outcomes for all OARs can be found in Table 3.

**Table 3:** Accumulated dose to the organs at risk, with and without APT strategy applied. Results denoted as median (min–max) or mean ± SD.

	Ipsilateral Parotid Dmean [Gy]		Contralateral Parotid Dmean [Gy]		Pharyngeal Constrictor Muscles Dmean [Gy]		Oral Cavity Dmean [Gy]	
Author (Year)	**Non-APT acc. dose**	**APT acc. dose**	**Non-APT acc. dose**	**APT acc. dose**	**Non-APT acc. dose**	**APT acc. dose**	**Non-APT acc. dose**	**APT acc. dose**
Bobić (2021) [9]	27.44 (13.63-57.90)	OAw: 23.80 (11.69-56.31) OAd: 23.60 (11.29-56.35)	19.84 (16.20-52.12)	OAw: 15.74 (14.48-52.82) OAd: 15.95 (14.21-52.81)	36.05 (10.73-59.84)	OAw: 30.86 (9.26-59.81) OAd: 30.90 (9.26-59.88)	12.25 (6.83-52.65)	OAw: 12.35 (6.18-50.95) OAd: 12.15 (6.21-51.20)
Minatogawa (2021) [10]	NA	PGR: 22.7 ± 7.2	NA	PGL: 35.8 ± 9.8	NA	Sup: 66.7 ± 3.0; Mid: 45.7 ± 11.5; Inf: 31.2 ± 7.1	NA	21.7 ± 6.2
Lalonde (2021) [11]	25.05 (19.33-56.40)	aRO: 22.43 (18.37-57.00) OA: 23.60 (11.29-56.35)	21.43 (18.41-28.76)	aRO: 19.85 (18.68-26.14) OA: 15.78 (14.21-26.85)	37.33 (14.26-60.33)	aRO: 38.01 (16.41-60.50) OA: 30.90 (9.26-59.88)	15.90 (8.38-50.82)	aRO: 17.85 (11.08-56.55) OA: 12.15 (6.21-51.20)
Nesteruk (2021) [12]	PGR: 18.7 (12.5-56.0)	PGR: 18.7 (13.1-55.2)	PGL: 18.4 (10.6-52.0)	PGL: 18.6 (10.4-52.1)	29.7 (8.9-59.5)	29.7 (8.6-59.6)	NA	NA
Yang (2020) [13]	36.05 ± 9.04	2^nd^ APT: 33.49 ± 6.67 1^st^ APT: 34.46 ± 6.84 aRO: 33.71 ± 6.80	PGL: 32.82 ± 5.81	2^nd^ APT: 32.56 ± 8.31 1^st^ APT: 31.72 ± 7.83 aRO: 31.07 ± 8.70	NA	NA	NA	NA
Cubillos-Mesías (2019) [14]	23.05 (19.21-56.76)	aRO: 21.74 (17.79-55.58)	19.93 (16.33-25.54)	aRO: 19.77 (10.61-23.28)	50.08 (39.47-63.64)	aRO: 50.80 (40.23-63.83)	39.62 (19.58-65.40)	aRO: 39.96 (19.34-65.43)
Botas (2018) [15]	NA	NA	NA	NA	NA	NA	NA	NA
Wu (2017) [16]	PGR: 7.64	PGR: 7.26	PGL: 8.73	PGL: 8.75	NA	NA	5.82	5.35
Kurz (2016) [17]	Similar as planned*	No reduction* Overall, no remarkable improvement	Similar as planned*	No reduction* Overall, no remarkable improvement	NA	NA	NA	NA
Simone (2011) [18]	32.90	29.80	19.50	18.30	NA	NA	NA	NA

	**Larynx**		**Spinal cord**		**Brainstem**	
	**Dmean [Gy]**		**Dmax [Gy]**		**Dmax [Gy]**	
**Author (Year)**	**Non-APT**	**APT**	**Non-APT**	**APT**	**Non-APT**	**APT**
	**acc. dose**	**acc. dose**	**acc. dose**	**acc. dose**	**acc. dose**	**acc. dose**
**Bobić (2021)**[9]	28.43 (7.01-42.13)	OAw: 24.48 (6.82-36.09)	D1cc: 16.65 (10.60-29.89)	OAw, D1cc: 11.99 (8.39-25.18)	D1cc: 1.19 (0.47-24.71)	OAw, D1cc: 0.97 (0.44-15.12)
		OAd: 24.37 (6.92-35.89)		OAd, D1cc: 12.03 (8.50-25.26)		OAd, D1cc: 0.99 (0.43-15.08)
**Minatogawa (2021)**[10]	NA	SGL: 25.5±8.6; GL:15.3±6.3	NA	D0.03cc: 42.7±3.2	NA	51.1±4.7
**Lalonde (2021)**[11]	33.20 (10.95-41.39)	aRO: 31.44 (13.96-41.39)	D1cc: 23.74 (18.58-30.81)	aRO: 25.78 (21.00-32.08)	D1cc: 5.04 (0.47-17.11)	aRO: 4.97 (0.60-29.07)
		OA: 24.37 (6.92-35.89)		OA: 12.03 (8.50-25.26)		OA: 0.99 (0.43-15.08)
**Nesteruk (2021)**[12]	23.6 (5.9-36.1)	23.1 (6.4-35.0)	D1cc: 12.2 (6.8-24.6)	D1cc: 12.4 (7.3-24.0)	NA	NA
**Yang (2020)**[13]	43.70 ± 4.39	2^nd^ APT: 40.21 ± 4.95	D2: 36.79 ± 5.58	2^nd^ APT, D2: 36.27 ± 3.65	D2: 45.36 ± 7.80	2^nd^ APT, D2: 45.53 ± 3.11
		1^st^ APT: 39.97 ± 5.30		1^st^ APT, D2: 36.44 ± 3.43		1^st^ APT, D2: 45.20 ± 3.01
		aRO: 34.62 ± 4.68		aRO, D2: 39.08 ± 4.16		aRO, D2: 44.75
**Cubillos-Mesías (2019)**[14]	40.10 (26.93-69.81)	aRO: 40.13 (27.08-69.91)	D1cc: 26.17 (11.42-31.93)	aRO, D1cc: 24.51 (11.17-32.75)	D1cc: 12.26 (0.41-23.95)	aRO, D1cc: 11.85 (0.75-23.74)
**Botas (2018)**[15]	Similar to the original plan	Similar to the original plan	NA	NA	NA	NA
**Wu (2017)**[16]	NA	NA	Dmax: 10.95	Dmax: 10.58	Dmax: 10.15	Dmax: 9.80
**Kurz (2016)**[17]	NA	NA	D2: 1 patient similar as planned. 1 patient increased from 42 to 47 Gy*	D2: 1 patient similar as planned. 1 patient decreased to 45 Gy*	NA	Overall, no remarkable improvement
				Overall, no remarkable improvement		
**Simone (2011)**[18]	35.3	31	Dmax: 42.1	Dmax: 41.7	Dmax: 44.8	Dmax: 42.2

APT: adaptive proton therapy, acc: accumulated, Dmean: mean dose, Dmax: max dose, D2: dose received by 2 % of the volume, D1cc: dose received by 1 cc of the volume, aRO: anatomical robust optimization, OA: online adaptation, OAw: weekly online adaptation, OAd: daily online adaptation, NA: not applicable, PGR: right parotid gland, PGL: left parotid gland, SGL: supraglottic larynx, GL: glottic larynx.

* Individual results for 2 representative patients.

#### Anatomical robust optimization approach

Three studies investigated an anatomical robust optimization approach (aRO) and compared this to their other (non)-APT strategies.[11], [13], [14] In this aRO method, in addition to the planning-CT, the first adaptive-CT [13] or the first two repeat-CTs [11], [14] were used together during plan optimization, which represent realistic anatomical variation. During plan optimization, this additional anatomical information was incorporated as additional scenarios during robust optimization.

Target coverage and sparing of some of the OARs in the aRO plans were found to be superior as compared to classical robust optimization [11], [13], [14], and even comparable to two plan adaptations, but with slight overdosage [13]. In [11], the clinical objectives for target coverage were met in 8/10 patients with this aRO technique, which was observed in only four patients for the non-adapted plans. In [14], target coverage did not fulfill the clinical goal in 5/20 patients in the non-adapted plans, which occurred only in one patient with the aRO strategy. These results indicate that including more CTs during plan optimization could reduce the frequency of plan adaptation. Nevertheless, daily APT outperformed the aRO approach since the target coverage goal was achieved in all ten patients.[11].

#### One or two APT interventions

Five studies investigated dosimetric effects of a single plan adaptation [10], [13], [16], [17], [18] and one study compared this with two times APT [13]. In Wu et al., a single APT intervention was performed in week 4 and dosimetric measures were calculated for the last 9 fractions. Wilcoxon signed rank test revealed significant dosimetric increases of the target D99 and D95 in the APT plans (p ≤ 0.04) as compared to the non-adapted accumulated plans, whereas mean doses to the right parotid and oral cavity were significantly decreased with APT (p = 0.03).[16] In Kurz et al. [17], reduced overdosing in the high- and low dose targets were observed after one APT. In Simone et al. [18], target prescription dose remained covered in non-adapted and APT plans. Average dose reductions in spinal cord, mandible, and larynx with APT were significant as compared to non-adaptive IMPT.

Yang. et al. [13] compared the dosimetric effect of a single APT to two times APT. The calculated dose to targets and OARs on the second APT-CT were compromised when none or single APT was applied, which indicated the need for additional plan adaptation. However, for the OARs, this was not observed in the accumulated dose. Due to anatomic changes between the planning-CT and second APT-CT, significant cold spots were observed in multiple patients, which were still visible in one patient on the CT of the second APT after one plan adaptation was performed. Accumulated dose D98% to the 63 Gy CTV (not shown in Table 2) was compromised after the first adaptation with mean D98% of 97.81 % and restored to 101.37 % after the second APT was applied.

Overall, when one plan adaptation was performed, all studies [13], [16], [17], [18] showed improved target coverage, but Yang et al. [13] showed that in their study, a second APT was required for target dose restoration in the low-dose CTVs. Also, differences in CTV D98 between one and two plan adaptations in the low-dose CTVs were statistically significant.[13].

#### Daily (online) vs. weekly APT

Other studies investigated the dosimetric effects of plan adaptations on a more frequent base, e.g., daily APT [9], [11], [12] or weekly plan adaptations [9], [15] were investigated.

The three included studies in this review on daily online APT showed on average an improved target coverage (D98, accumulated dose) for the high- and low risk CTVs compared to the original plan, up to 2.5 Gy (3.6 %) and 1.7 Gy (3.0 %) respectively, see Table 2. Overall, dose to OARs was reduced with daily APT, especially in the PCMs and larynx notable reductions were observed with daily adaptive strategy.[9], [11], [12] Compared to weekly APT, Bobić et al. [9] found that for individual fractions, daily APT yielded better target coverage, since in weekly APT the target coverage fluctuated. These fluctuations were probably caused by daily positioning errors and anatomical variations. On average, target coverage was comparable in weekly and daily APT. Also, OAR dose reductions in daily and weekly APT were comparable, see Table 3 for the summarized results.[9].

### Frequency and timing of APT

With one adaptative intervention, the largest improvement in target coverage was found, and an eventual second adaptation improved the target coverage further. Both daily and weekly APT were able to improve target coverage, while daily APT outperformed weekly APT for individual fractions. OAR dose reductions were comparable between daily and weekly APT.[9].

In [17], [18], the moments for APT interventions in the computer simulations were taken the same as those used in clinical photon IMRT treatments of the patient, while in [13], [16] the need for APT was clinically decided during IMPT treatment. The moment of APT in these last two mentioned studies ranged between week 2 and 4, see Table 1. In [13], there was clinically-two times an APT required during the RT course. The moment of this second APT approach was in the range of 12–17 fractions after the first APT.

## Discussion

In this review, ten studies published between January 2010 and March 2022 were identified for addressing the dosimetric impact of an APT strategy in HNC IMPT versus no plan adaptation.

APT in HNC patients lead to an improved target coverage and similar or improved OAR doses. In case no plan adaptation was performed, target coverage deteriorated below the clinical goals, which was restored after APT was applied. OAR doses remained below the clinical constraints during the RT course, and after APT was applied, the dose remained similar or decreased. No evidence for the optimal timing to perform APT can be obtained from this review. A single APT was in most cases sufficient to restore target coverage, but there was not one specific optimal moment to perform APT. Weekly APT was on average able to maintain target coverage while daily APT could reach better target coverage compared to weekly APT for individual fractions.

We found that APT was indicated due to target coverage deterioration during the RT course, and doses to OARs were never the trigger for an APT intervention.[9], [11], [13], [14], [16], [17] While in accordance with earlier APT studies from de Orneleas [20] and Stützer [21], this finding contrasts with some studies on (A)RT treatments with photons, where violated OAR constraints were the main triggers for plan adaptations, and to a lesser extent target coverage deterioration.[22], [23] On the other hand, the large international POP-ART survey on ART in photon therapy revealed that in most centres, both target and OAR doses could be a reason for plan adaptation.[24] Although PTV-based planning is generally applied for photon therapy and robust optimization methods are applied to proton therapy, in this review the majority (7/10) of the studies were not based on robust optimization methods. Therefore, the contrast in ART indication between photons and protons in this review is not likely to be caused by the difference in optimization methods. The difference can possibly be explained by the superior initial OAR sparing with IMPT, and IMPT even has lower dose to OARs as compared to adapted photon RT plans, as observed in [18]. Thus, since OAR doses in IMPT generally remain below constraints; indication for APT should be derived from target dose degradation. The observed dose reductions in OARs could potentially reduce the normal tissue complication probability (NTCP) and might be clinically relevant. However, none of the studies reported on clinical outcomes. Therefore, this should be investigated in future clinical studies on APT where toxicity and NTCP should also be considered and measured.

Different moments and frequencies of APT were reported, and no conclusions on the most appropriate timing for APT can be drawn from this review. This was also found in [25], where replanning occurred during all weeks of IMPT, but mostly in week 2 (26 %). This finding is in accordance with photon ART in HNC, where the optimal timing is not well defined, but seems to be early, i.e., during the first two weeks of the treatment.[23] For IMPT, mostly-one plan adaptation was sufficient to restore the target goals with similar dose to OARs, and in some HNC patients, a second adaptation may still be required after one adaptation is already performed as seen in [13].This marks the need to continue monitoring the plan quality during the RT course, also after a plan adaptation was already performed.

When comparing to weekly and daily plan adaptations, both APT strategies can reach higher target coverage compared to the non-adapted cumulative dose with overall a lower dose to OARs [9], [11], [12], where daily APT yielded better target coverage on individual fractions.[9] Despite the daily APT strategy was planned without robustness margins, target coverage was maintained with daily online adaptation.[9], [11], [12] Since daily APT offers possibilities for planning with only robustness settings for range uncertainties, this allows for dose reductions in the OARs, which was shown by [9], [11]. In addition, the need for replanning, and thus the APT frequency, depends on how precise the planned dose needs to be maintained during the RT course, as was found by Stoll et al. [26]. Also, when no robustness settings are applied in planning, it is expected to be more sensitive to anatomical changes and thus to require more, or even daily, plan adaptations.

An alternative for re-planning APT strategies can be anatomical robust optimization, where multiple CTs are incorporated during plan optimization.[11], [13], [14] With this aRO approach, the need for APT can in principle be reduced, which could make it attractive to incorporate in the RT planning workflow. However, potential anatomical variations are generally unknown during planning, as re-imaging is normally performed while the fractionated treatment has already started. Therefore, a prediction model for anatomical changes could be helpful for clinical introduction of this aRO approach, which should preferentially be patient-specific since anatomical variations differ a lot between patients. Moreover, large unpredictable anatomical changes might still occur, where the aRO approach could be not robust enough for. Such cases would still require an additional plan adaptation. This was observed in [11], where daily adaptation outperformed the aRO strategy regarding target coverage. Van de Water et al. [27] performed a similar aRO study, with incorporating nasal cavity filling during IMPT planning for sinonasal tumors. They also found that their aRO approach outperformed the non-adapted plans with adequate target coverage and reduced OAR doses, but that online daily adaptation could reduce the dose to OARs even further.

One of the limitations of this study is that the number of included studies in this review is small, and since most of them were based on simulations, more clinical APT studies are needed. Moreover, the patient numbers of the included studies are small, i.e. only one study investigated more than 10 patients, [14]. Therefore more research in larger patient groups is needed to draw conclusions on the added value and optimal timing of APT for HNC patients. Furthermore, since systematic comparisons between the different methods and timing or frequency of APT have been barely performed, future research is required. Additionally, half of the studies in this review performed the dose re-calculations on CBCT, which is known to be challenging. Due to inferior image quality of CBCT, segmentation of targets and OARs is difficult, and inaccuracy of the reconstructed Hounsfield Units affect the dose calculation. Therefore, the studies used scatter corrections such as validated by [28], [29]. Next to this, the studies included in this review were partly based on included HNC patient cases which clinically needed a plan adaptation, either in IMRT or IMPT. Therefore, the result that target coverage deteriorated below the clinical goals during the IMPT course, and thus requiring an APT, may not be applicable for all HNC patients. Indeed, in [18], it was observed that target coverage remained adequate in the non-adapted IMPT plans and APT was not necessarily indicated for these patients. Consequently, not all HNC patients require APT during IMPT and the dosimetric benefits found in this review may overestimate the general benefit of APT. While Deiter et al. [25] found that 63 % of the HNC patients needed IMPT replanning, Stanforth et al. [30] found that approximately 30 % of their HNC cohort were replanned during IMPT. In accordance with this review, they found that in most cases one plan adaptation was sufficient to restore target coverage, and in some cases two plan adaptations were necessary. In spite of these findings, it seems that the number of patients in photon therapy that need a plan adaptation is lower compared to IMPT. Jensen et al. [22] found that about 21 % of the HNC patients required replanning at least once during the IMRT course.

APT is required to improve and restore target coverage in IMPT in HNC, but incorporating APT into the clinical workflow poses some challenges. The clinical workflow for APT includes imaging, re-contouring, plan(re-)calculation and plan evaluation, which makes APT time- and resource intensive. Monitoring anatomical changes and target dose deteriorations to determine the timing when APT is needed, regular re-imaging has a prominent role. This workflow can be aided with efficient decision tools, such as thresholds that trigger APT. Besides, automation of the workflow can make the application of APT more efficient. Several studies are ongoing in the field of auto-segmentation of the targets [31], [32] and OARs [33], [34] using artificial intelligence. This can make the delineation faster and more consistent. Furthermore, the development of automatic dose optimizations, which are necessary to make fast and accurate APT possible, are part of ongoing studies [35], [36].

With automation of the APT workflow, the adapted IMPT plans may be produced with a consistent high quality and produce a new adapted plan within a short timeframe to be able to anticipate on the needed adaptation and apply this as fast as possible, even for online daily application. With this workflow, the patient will be treated with a maximally individualized IMPT plan, adjusted to the specific anatomical positions at that day, and hence maximal beneficial effects of IMPT sparing can be obtained. Also, daily online adaptation may provide possibilities in accepting target underdosage, while sparing critical OARs at a specific daily fraction. Future research into this this field has to investigate on the additional benefits of this planning strategy.

## Conclusion

APT during IMPT course in HNC patients leads to restored target coverage in case of deteriorations caused by set-up or patient anatomy variations. The largest improvement in target coverage was found with one plan adaptation, and an eventual second or more frequent adaptation improved the target coverage further. With APT, OAR doses remained generally equal or reduced additionally. Online daily APT is the most robust for individual fractions, but weekly APT seems also sufficient for target coverage restoration. The most optimal timing for APT e.g., a specific week, is yet to be determined.

## Funding Statement

The research for this work was funded by Varian, a Siemens Healthineers Company (HollandPTC-Varian Consortium grant ID 2019021), and partly financed by the Surcharge for Top Consortia for Knowledge and Innovation (TKIs) from the Dutch Ministry of Economic Affairs and Climate.

## Declaration of Competing Interest

The authors declare that they have no known competing financial interests or personal relationships that could have appeared to influence the work reported in this paper.
